# Ceramides: a potential cardiovascular biomarker in young adult childhood cancer survivors?

**DOI:** 10.1093/ehjopen/oeae026

**Published:** 2024-04-04

**Authors:** Olof Broberg, Constance G Weismann, Ingrid Øra, Thomas Wiebe, Reijo Laaksonen, Petru Liuba

**Affiliations:** Department of Pediatric Cardiology, Skåne University Hospital, Lasarettgatan 48, SE-221 85 Lund, Sweden; Department of Clinical Sciences, Pediatrics, Lund University, Lasarettgatan 40, SE-221 45 Lund, Sweden; Department of Pediatric Cardiology, Skåne University Hospital, Lasarettgatan 48, SE-221 85 Lund, Sweden; Department of Clinical Sciences, Pediatrics, Lund University, Lasarettgatan 40, SE-221 45 Lund, Sweden; Department of Pediatric Cardiology and Pediatric Intensive Care, Ludwig-Maximilian University, Klinikum Grosshadern, Marchioninistr. 15, DE-81377 Munich, Germany; Department of Clinical Sciences, Pediatrics, Lund University, Lasarettgatan 40, SE-221 45 Lund, Sweden; Department of Pediatric Oncology, Skane University Hospital, Lasarettgatan 48, SE-221 85 Lund, Sweden; Department of Clinical Sciences, Pediatrics, Lund University, Lasarettgatan 40, SE-221 45 Lund, Sweden; Department of Pediatric Oncology, Skane University Hospital, Lasarettgatan 48, SE-221 85 Lund, Sweden; Finnish Cardiovascular Research Center, Tampere University, Arvo Ylpön Katu 34, P.O. Box 100, FI-33014, Finland; Zora Biosciences, Biologinkuja 1, FI-02150 Espoo, Finland; Department of Pediatric Cardiology, Skåne University Hospital, Lasarettgatan 48, SE-221 85 Lund, Sweden; Department of Clinical Sciences, Pediatrics, Lund University, Lasarettgatan 40, SE-221 45 Lund, Sweden

**Keywords:** Ceramides, Childhood cancer survivors, Biomarker

## Abstract

**Aims:**

The aim of this study was to investigate circulating ceramides involved in cardiovascular disease (CVD) in young adult childhood cancer survivors (CCS) and their correlations to previously reported adverse cardiovascular changes in this cohort.

**Methods and results:**

Fifty-seven CCS and 53 healthy controls (age 20–30 years) were studied. Plasma long-chain ceramides, known to be cardiotoxic (C16:0, C18:0, C24:0, and C24:1), were analysed by mass spectrometry. The coronary event risk test 2 (CERT2) score was calculated from the ceramide data. Cardiac and carotid artery ultrasound data and lipid data available from previous studies of this cohort were used to study partial correlations with ceramide and CERT2 score data. All four analysed ceramides were elevated in CCS compared with controls (*P* ≤ 0.012). The greatest difference was noted for C18:0, which was 33% higher in CCS compared with controls adjusted for sex, age, and body mass index (BMI) (*P* < 0.001). The CERT2 score was higher in CCS compared with controls (*P* < 0.001). In the CCS group, 35% had a high to very high CERT2 score (7–12) when compared with 9% in the control group (*P* < 0.001). The CCS subgroup with a CERT2 score ≥ 7 had higher heart rate, systolic blood pressure, and higher levels of apolipoprotein B compared with CCS with a CERT2 score < 6 (*P* ≤ 0.011). When adjusted for age, sex, and BMI, CERT2 score was significantly correlated with arterial stiffness, growth hormone, and cranial radiotherapy (*P* < 0.044).

**Conclusion:**

Ceramides could be important biomarkers in understanding the pathophysiology of CVD and in predicting CVD disease risk in young adult CCS.

## Graphical Abstract

**Graphical Abstract:**
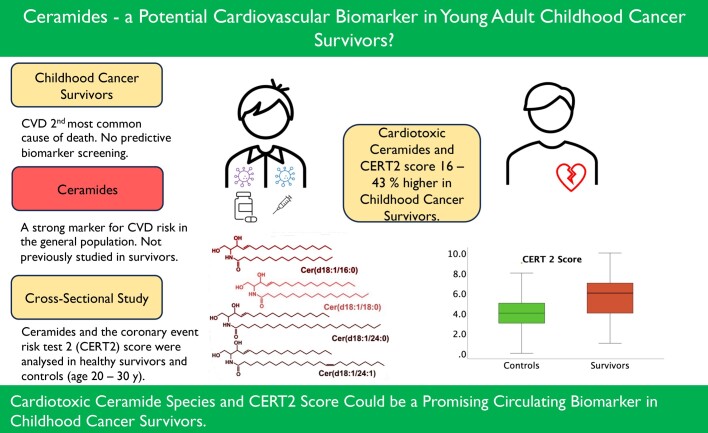

## Introduction

Ceramides belong to the group of sphingolipids and are essential components of cell membrane structure and function.^1^ Ceramide accumulation has been suggested to be important in cancer treatment efficacy.^2^ More recently, ceramides have been suggested to be a key messenger in the onset and progression of cardiovascular disease (CVD) and an independent predictor for major cardiovascular events (MACE) in older adults in the general population.^3^

In animal models, enzymatic inhibition of ceramide production inhibited or reversed cardiomyopathy and atherosclerosis development.^4,5^ In heart failure patients, specific subtypes of circulating ceramides (C16:0—C24:1) were elevated in the blood, but were normalized after ventricular unloading therapy, suggesting that these ceramide subtypes may provide important new targets in monitoring heart failure treatment.^6^ Because ceramides are considered potential drivers of CVDs, ceramide-lowering therapies are also suggested for both primary and secondary prevention strategies.^7^

Long-chain ceramides (such as C16:0–C24:1) have been implicated in the pathophysiology of inflammation, cardiac remodelling, endothelial dysfunction, and atherosclerosis, all of which being important mechanisms in the development of CVD.^8^ The coronary event risk test 2 (CERT2) is based on different circulating long-chain ceramides and has emerged as a promising tool for 10-year CVD risk prediction of non-fatal and fatal cardiovascular events in both healthy adults and in patients with established CVD.^9–11^

Childhood cancer survivors (CCS) are a unique population with a high prevalence of cardiometabolic risk factors such as dyslipidaemia, hypertension, and physical inactivity and with a significantly elevated risk for heart failure and myocardial infarction compared with the general population.^12,13^ The increased occurrence of sarcopenic obesity and dyslipidaemia in CCS could possibly increase ceramide production and promote the accumulation of ceramides into cardiovascular tissues.^14,15^ In addition, various cardiotoxic treatment regimens including radiotherapy (RT) and chemotherapy, which many CCS typically require, may further increase the cardiovascular risk in CCS.^16^

To the best of our knowledge, circulating ceramides have not previously been studied in CCS. Determining circulating ceramides associated with CVDs in CCS might explain some of the increase in cardiovascular risk in CCS. Ceramides might also help to further improve cardiovascular risk assessment in this CCS. The aim of this study was to determine circulating cardiotoxic ceramides and the CERT2 score in a cohort of young adult CCS, without CVD, and to compare them with healthy controls. The second aim was to study correlations of circulating ceramides to certain subclinical adverse changes in the cardiovascular system that we have earlier reported in this cohort.

## Methods

This was a single-centre cross-sectional cohort study of circulating ceramide species, known to be associated with CVD, in young adult Swedish CCS that previously had undergone treatment at the Pediatric Oncology Centre at the Skåne University Hospital in Lund, Sweden. All CCS were identified from the Registry for Childhood Malignancies in Southern Sweden (BORISS).^17^ The inclusion criteria for the study were (i) cancer diagnosis under the age of 18, (ii) survival more than 5 years after the disease remission, and (iii) age between 20 and 30 years. Exclusion criteria were (i) brain tumour diagnosis, (ii) previous or current CVD or any cardiovascular complication during cancer treatment, (iii) any current chronic disease or syndrome, and (iv) pregnancy.

**Figure 1:**
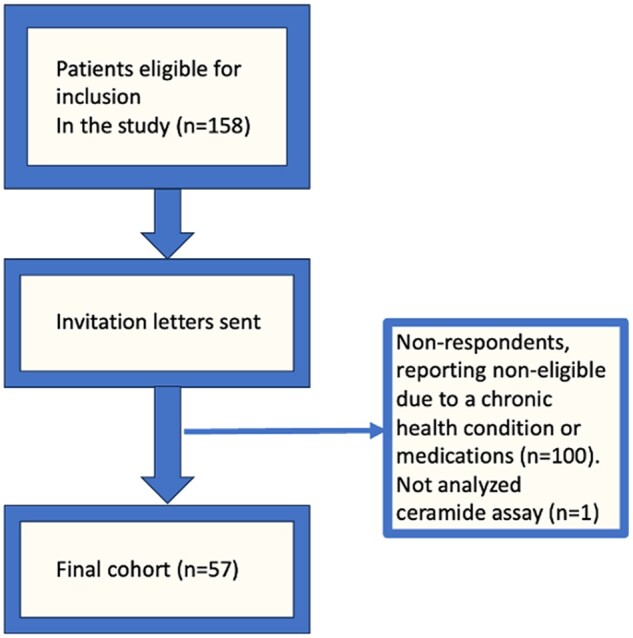
Recruitment of childhood cancer survivors.

One hundred and fifty-eight CCS met the eligibility criteria and received a written invitation to participate. Twenty-six reported that they had a chronic health condition and/or were on medication and 74 did not respond (*Figure 1*). The ceramide assay was not analysed in one CCS. The final cohort consisted of 57 CCS. Fifty-three healthy controls with similar sex and age were recruited via written announcements at the Skåne University Hospital area in Lund, Sweden. Both cohorts were previously screened for cardiac morphology and function, cardiac reserve and different lipid and apolipoprotein biomarkers, as well as large-scale proteomic study of different circulating cardiovascular proteins.^18–20^ Informed written consent was obtained from all study participants. The study protocol was approved by Lund University’s Regional Ethical Committee for Human Research (DNR 2013/205).

### Clinical, cardiac, and carotid artery assessments

Data regarding treatment with anthracyclines (including the cumulative dose), cranial, and mediastinal RT were collected from BORISS.^17^ The cardiac and carotid artery assessments were described in detail in our previous study on this cohort.^18,19^ Blood pressure (BP) was measured in the right arm. Weight and height were measured, and body mass index (BMI) was calculated. Overweight was defined as BMI > 25 kg/m^2^.

Echocardiographic measures of cardiac systolic and diastolic function were left ventricular ejection fraction (LVEF) calculated by the Simpson bi-plane method of discs LVEF, global longitudinal strain (GLS), and tissue Doppler-derived diastolic velocities (*é*). Left ventricular posterior wall thickness in diastole (LVPWd) was also measured. Tricuspid annular plane systolic excursion (TAPSE) was used as a measure for right ventricular systolic function.

Carotid artery ultrasonography was used to assess common carotid artery (CCA) distensibility index (DI) and stiffness index (SI) and carotid intima-media thickness (CIMT) as previously described.^18^

### Ceramide analysis and coronary event risk test 2-score

Morning fasting blood samples were collected at the same visit as collection of data on clinical and cardiovascular measures and were frozen to −80°C. All samples were later shipped as a whole batch for ceramide analysis and CERT1 and CERT2 score calculation (Zora Biosciences, Espoo, Finland). Plasma ceramides were analysed by mass spectrometry, in multiple reaction monitoring (MRM) mode. Quantification was assessed through calibration line samples constructed with known amounts of synthetic ceramides (d18:1/16:0, d18:1/18:0, d18:1/24:0, and d18:1/24:1, here named C16:0, C18:0, C24:0, and C24:1 for convenience) and corresponding standards. Included in the analysis were also phospholipids [PC(14:0/22:6), PC(16:0/16:0), and PC(16:0/22:6), here named PC26:6, PC16:0, and PC22:5 for convenience].

Peak area ratios of analysed ceramides and phospholipids, to its corresponding deuterated form, were calculated and plotted against the known added synthetic ceramide concentrations and finally linear regression analysis. Plasma ceramide concentrations were then derived from the obtained individual regression equations. The final ceramide and phospholipid concentrations in plasma were presented in picomoles/litre (pM/L).

### Other analyses

Analyses for lipids and apolipoproteins, high-sensitivity C-reactive protein (CRP), and N-terminal-pro-brain natriuretic peptide (NT-pro-BNP) are previously described and reported.^18^ Relative growth hormone (GH) concentration, expressed in Normalized Protein eXpression (NPX) units, was previously determined.^20^

### Statistics

Normally distributed variables were expressed as the mean ± standard deviation (SD). Categorical variables were expressed as numbers and percentages (%). The primary outcome variables were the ceramides and the CERT2 score. Secondary outcome measures that were used as possible markers for ceramide-induced cardiac remodelling were CCA, DI and SI, left ventricular GLS, LVEF, TAPSE, tissue Doppler-derived early diastolic *é*-wave velocity, and LVPWd indexed to body surface area (LVPWd/BSA). Lipid cardiovascular biomarkers [LDL, total cholesterol (TC), and triglycerides (TG)], apolipoprotein cardiovascular biomarkers [apolipoprotein B (Apo-B) and the Apo-A/Apo-B ratio] and clinical measures were also included. Carotid intima-media thickness was also included as a secondary measure in the analysis but was not previously reported to be different in CCS compared with controls.

Differences in clinical measures were calculated by Student’s *t*-test for continuous variables and by χ^2^-test for dichotomous variables. If the data were non-normally distributed, non-parametric testing with Mann–Whitney *U* test was performed. Differences in mean values of different ceramides (C16:0, C18:0, C24:0, and C24:1), and CERT1 and CERT2 score between CCS and controls were assessed by analysis of co-variance, adjusting for sex, age, and BMI (analysis of covariance [ANCOVA]). Based on CERT2 score, CCSs were further divided into two groups: low-to-moderate score (0–6), and high-to-very-high (7–12) CERT2 score according to previously published risk-score charts.^21^ Differences between these two groups for clinical outcome measures were assessed by ANCOVA adjusted for the cumulative anthracycline (AC) dose, and cranial and mediastinal RT.

In the CCS cohort, we performed partial correlations of analysed ceramides to the above-mentioned cardiovascular measures as well as the cumulative AC dose, exposure to cranial and mediastinal RT, BMI, heart rate (HR), CRP, and NT-pro-BNP and BP, adjusting for sex, age, and BMI. All statistical analyses were done with SPSS 28.0 (IBM, NY, USA). *P*-values <0.05 were regarded as significant.

## Results

A summary of the different childhood diagnoses and treatments in the CCS group is shown in Supplementary material online, *S1*. Previously reported clinical data, cardiovascular measures, and circulating lipid and apolipoprotein biomarkers in CCS and controls can be found in *Table 1*. None of CCS nor controls had any previous or current CVD or were on any cardiovascular or lipid-lowering medications. There was a small but significant difference in age between CCS and controls [mean age, years, (SD); controls 24.4 years (2.4) and CCS 25.4 years (2.5)].

**Table 1:** Previously reported clinical and cardiovascular measures

Variable	CCS (mean, SD), *n* = 57	Controls (mean, SD), *n* = 53	*P*-value
Age (years)	25.36 (2.47)	24.40 (2.40)	**0.041**
Sex (females, %)	23 (40.35)	18 (33.96)	ns
BMI (kg/m^2^)	24.40 (3.47)	24.70 (3.71)	ns
Overweight (*n*, %)	20 (35.0%)	16 (30.8%)	ns
Systolic BP (mmHg)	118 (11.22)	118 (11.69)	ns
Diastolic BP (mmHg)	75 (8.23)	73 (5.97)	ns
LDL (mM)	2.81 (0.75)	2.21 (0.78)	**<0.001**
TG (mM)	1.14 (0.76)	0.78 (0.34)	**0.002**
TC (mM)	4.51 (0.85)	3.83 (0.82)	**<0.001**
Apo-B (g/L)	0.82 (0.21)	0.72 (0.20)	**<0.001**
Cystatin C (mg/L)	0.88 (0.14)	0.83 (0.10)	**0.032**
GFR (mL/min/1.73 m^2^)	98.22 (17.46)	104.10 (13.13)	**0.050**
CRP over detection limit (*n*, %)	33 (58.9%)	40 (75.5%)	ns
High TnT (*n*, %)	0 (0.0%)	2 (3.8%)	ns
High NT-pro-BNP (*n*, %)	6 (10.5%)	1 (1.9%)	ns
ccaDI (%/10mmHg)	2.61 (0.69)	3.47 (0.83)	**<0.001**
ccaSI (no unit)	4.62 (1.17)	3.35 (0.83)	**<0.001**
CIMT (mm)	0.46 (0.05)	0.44 (0.05)	ns
LVEF (%)	58.85 (4.97)	60.79 (4.85)	**0.014**
GLS (%)	−18.90 (2.14)	−20.35 (2.01)	**<0.001**
TDI *é* (cm/s)	12.64 (1.86)	14.63 (2.37)	**<0.001**
LVPWd/BSA (mm/m^2^)	3.79 (0.67)	4.30 (1.00)	**0.002**
TAPSE (mm)	24.99 (4.46)	27.80 (5.13)	**0.006**
*Treatment characteristics*			
Follow-up time (years)	16.41 (5.92)	—	—
Cum AC (mg/m^2^)	193.45 (107.29)	—	—
Cranial RT (*n*, %)	8 (14)	—	—
Mediastinal RT (*n*, %)	10 (17.5)	—	—
*Diagnosis*			
Leukaemia (*n*, %)	23 (40.4)	—	—
Lymphoma (*n*, %)	19 (33.3)	—	—
Wilms (*n*, %)	10 (17.5)	—	—
Sarcoma (*n*, %)	5 (8.8)	—	—

BMI, body mass index; BP, blood pressure; TG, triglycerides; TC, total cholesterol; Apo, apolipoprotein; Creat, creatinine; GFR, glomerular filtration rate; hsCRP, high sensitive C-reactive protein; NT-pro-BNP, N-terminal-pro-brain natriuretic peptide; TnT, troponin-T; CCA, common carotid artery; DI, common carotid artery distensibility index; SI, common carotid artery stiffness index; LVEF, left ventricular ejection fraction; GLS, global longitudinal strain; TDI *é*, tissue Doppler-derived *é*-wave velocity; LVPWd/BSA, left ventricular posterior wall diameter indexed to body surface area; TAPSE, tricuspid annular plane systolic excursion; Cum AC, cumulative anthracycline dose; RT, radiotherapy. *P*-values <0.05 were considered signifcant and are written in bold text.

Childhood cancer survivors lipid and apolipoprotein results were different from controls with higher LDL, Apo-B, Apo-B to A1 ratio, TG and TC levels [mean (SD); LDL (μM), CCS, mean 2.8 (0.7), controls mean 2.2 (0.8), TC (μM) CCS, mean 4.5 (0.9), controls mean 3.8 (0.8), Apo-B (g/L), CCS mean 0.9 (0.2), controls mean 0.7 (0.2), Apo-B/Apo-A1 ratio, CCS mean 0.6 (0.2), controls 0.5 (0.2), TG (μM), CCS mean 1.2 (0.8), controls 0.8 (0.4), *P* < 0.009 for all].

Left ventricular systolic function as shown by LVEF and GLS was lower in CCS compared with controls [LVEF, %, (SD); CCS 58.6 (5.0), controls 60.8 (4.9), *P* = 0.01, GLS, %, (SD); CCS −19.0 (2.1), controls −20.4 (2.0), *P* < 0.001]. Tricuspid annular plane systolic excursion as a measure of right ventricular systolic function was lower in CCS compared with controls [TAPSE, mm, (SD); CCS 25.2 (4.4), controls 27.8 (5.1), *P* = 0.005]. Further left ventricular posterior wall thickness indexed to body surface area was lower in CCS compared with controls [LVPWd/BSA, mm/m^2^ (SD); CCS 3.8 (0.7), controls 4.3 (1.0), *P* = 0.002].

Common carotid artery distensibility and SI (ccaDI and ccaSI) were different in CCS compared with controls: mean (SD), ccaDI (%/10 mmHg), CCS 2.6 (0.7), controls 3.5 (0.8), ccaSI, CCS 4.6 (1.2), controls 3.4 (0.8), *P* < 0.001 for both.

### Circulating ceramide concentrations and coronary event risk test 2 score

Ceramides samples were collected at a mean follow-up time after childhood cancer treatment of 16.4 (5.9) years. All analysed ceramides were higher in CCS compared with controls and the biggest difference was for C18:0 that was 33% followed by C24:1 and C16:0, respectively, that were 26 and 16% higher in CCS compared with controls adjusted for sex, BMI, and age (*P* < 0.001 for all). Neither sex nor age was significant covariates for any ceramides, but BMI was for C18:0, C24:0, and C24:1 (*P* < 0.008). The CERT2 score was significantly higher in CCS compared with controls [mean (SD) 5.7 (2.2) vs. 4.0 (2.0), *P* < 0.001]. The CERT1 score was also higher in CCS compared with controls [mean (SD) 4.4 (2.6) vs. 3.1 (2.0), *P* = 0.003]. Ceramide results were missing in one CCS and in none of the controls. Results are shown in *Table 2*. Further, the analysis of phospholipids and ratios of ceramides and phospholipids used to calculate the CERT1 and CERT2 scores are shown in Supplementary material online, *S2*.

**Table 2:** Different ceramides and coronary event risk test 2 score in childhood cancer survivors and controls

	CCS (*n* = 57), Mean (SD)	Control (*n* = 53), Mean (SD)	*P*-value
C16:0 (pmol/L)	0.22 (0.05)	0.19 (0.04)	**<0**.**001**
C18:0 (pmol/L)	0.08 (0.03)	0.06 (0.02)	**<0**.**001**
C24:0 (pmol/L)	2.29 (0.58)	2.02 (0.48)	**0**.**012**
C24:1 (pmol/L)	1.20 (0.34)	0.95 (0.27)	**<0**.**001**
CERT2 score (no unit)	5.70 (2.15)	3.98 (2.01)	**<0**.**001**

Comparison of different ceramides and CERT2 score between CCS and controls using ANCOVA, controlling for sex, BMI, and age. *P*-values <0.05 were considered signifcant and are written in bold text.

**Figure 2:**
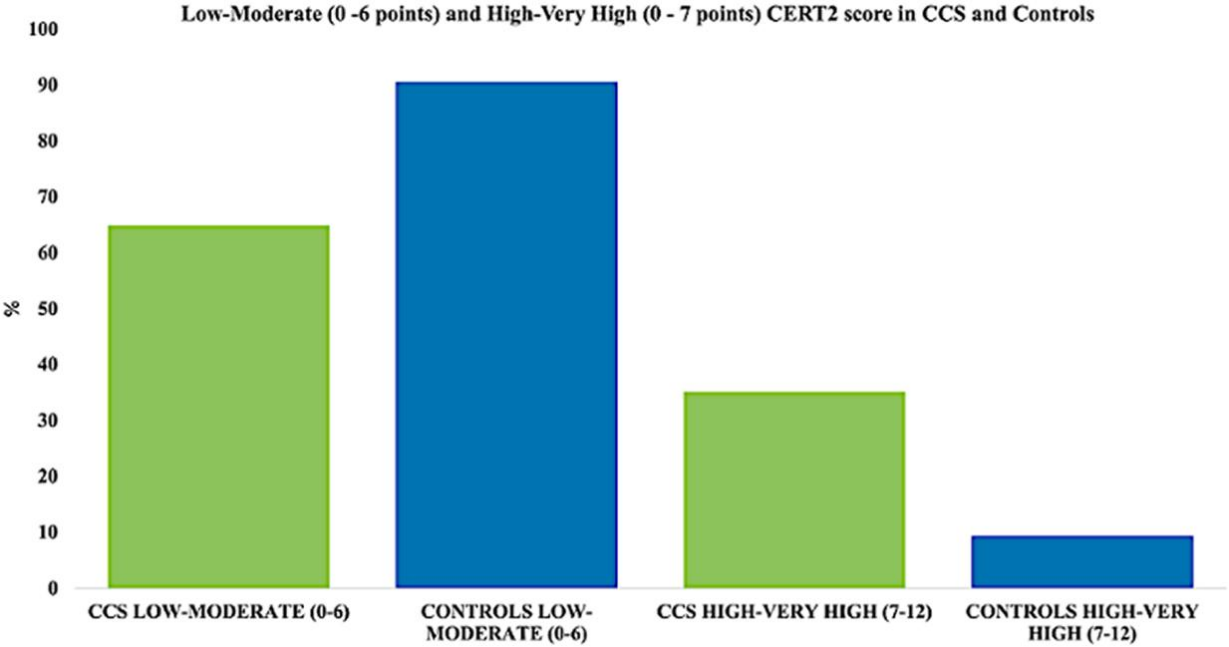
Histograms showing the distribution of low-to-moderate (0–6 points) and high-to-very high (0–7 points) coronary even risk test 2 (CERT2) score in childhood cancer survivors (CCS) and in controls; χ^2^  *P* = 0.001.

Based on CERT2 score, CCS and controls were divided into two groups: low-to-moderate score (0–6 points) and high-to-very-high score (7–12 points). Thirty-five per cent of CCS had high to very high CERT2 score compared with 9% of controls (χ^2^, *P* = 0.001). Coronary event risk test 2 group divisions are shown in *Figure 2*.

Cardiovascular measures, arterial stiffness, and lipid biomarkers in the CCS cohort that were known to be impaired in CCS as well as sex, age, BMI, heartrate, and systolic and diastolic blood pressure were compared among the CERT2 groups by ANCOVA adjusting for cardiotoxic treatments (*Table 3*). Systolic blood pressure (SBP) and HR was higher among CCS with a CERT 2 score ≥ 7 (*P* ≤ 0.003). Childhood cancer survivors with a CERT 2 score ≥ 7 had higher Apo-B and a higher Apo-B/Apo-A1 ratio (*P* ≤ 0.012) and had a lower carotid DI (*P* = 0.022). Triglycerides were 43% higher in CCS with a CERT 2 score ≥ 7, but the difference was not significant.

**Table 3:** Subgrouping of childhood cancer survivors by coronary event risk test 2 score and comparisons for cardiovascular and clinical measures between the subgroups adjusting for cardiotoxic treatments

	CERT2: 0–6	CERT2: 7–12	*P*-value
*n*, %	37 (65.00%)	20 (35.00%)	
Age (years)	25.71 (2.61)	24.80 (2.18)	ns
Sex (F, %)	14 (37.8%)	9 (45.0%)	ns
BMI (kg/m^2^)	23.98 (3.27)	24.44 (3.78)	ns
SBP (mmHg)	115.22 (8.54)	125.40 (12.73)	**<0.001**
DBP (mmHg)	74.65 (7.42)	78.35 (8.65)	ns
HR (b.p.m.)	69.03 (11.24)	76.60 (9.41)	**0.002**
LDL	2.69 (0.71)	3.03 (0.79)	ns
Total cholesterol	4.50 (0.90)	4.50 (1.40)	ns
TG	1.00 (0.48)	1.43 (1.08)	ns
Apo-B	0.80 (0.17)	0.95 (0.25)	**0.010**
Apo-B/Apo-A1	0.56 (0.14)	0.68 (0.20)	**0.011**
ccaDI (%/10mmHg)	2.78 (0.76)	2.33 (0.42)	**0.022**
ccaSI (no unit)	4.44 (1.17)	4.81 (1.01)	ns
CIMT (mm)	0.45 (0.05)	0.44 (0.06)	ns
LVPWd/BSA (mm/m^2^)	3.73 (0.90)	3.68 (1.07)	ns
LVEF (%)	57.92 (7.38)	58.76 (6.44)	ns
TDI *é* (cm/s)	12.48 (2.57)	12.01 (2.53)	ns
GLS (%)	−19.43 (3.65)	−18.91 (3.25)	ns
TAPSE (mm)	24.00 (7.75)	23.50 (4.75)	ns

Childhood cancer survivors and controls divided in subgroups by low–moderate (0–6) and high–very high (0–7) coronary event risk test 2 (CERT2) score. Comparison between subgroups of cardiovascular variables knows to be different between CCS and controls. Variables are shown with the mean (SD). ANCOVA, adjusting for the cumulative AC dose and cranial and mediastinal RT, was used for subgroup comparisons between CCS with a CERT2 score of 7–12 and CCS with a CERT2 score of 0–6.

BMI, body mass index; SBP, systolic blood pressure; DBP, diastolic blood pressure; HR, heart rate; Apo, apolipoprotein; CERT2, coronary risk even test 2; DI, common carotid distensibility index (mean of the right carotid artery and CCA); SI, common carotid artery (mean of the right carotid artery and CCA); CCA, common carotid artery; CIMT, carotid intima-media thickness; LVPWd/BSA, left ventricular posterior wall thickness in the diastole indexed to body surface area; GLS, global longitudinal strain; TDI *é*, tissue Doppler-derived early diastolic *é*-wave; TG, triglycerides. *P*-values <0.05 were considered signifcant and are written in bold text.

### Correlations of ceramides to clinical and cardiovascular measures

The data are shown in *Table 4*. Ceramides and CERT2 score were analysed by partial correlations with previously reported clinical characteristics (including the cumulative AC dose and exposure cranial or mediastinal RT) and cardiovascular measures in the CCS cohort after adjustment for sex, BMI, and age.

**Table 4:** Partial correlations (*r* and *P*-values) of the measured ceramides and the coronary event risk test 2 score to different clinical and cardiovascular measures controlling for age, sex, and body mass index in the childhood cancer survivors cohort (*n* = 57)

	C16:0	C18:0	C24:0	C24:1	CERT2 score
	***r* (*P*-value)**				
BMI (kg/m^2^)	0.18 (ns)	**0.37 (0.005)**	0.26 (ns)	0.22 (ns)	0.06 (ns)
HR (b.p.m.)	**0.36 (0.008)**	**0.28 (0.038)**	0.20 (ns)	**0.35 (0.010)**	**0.36 (0.008)**
SBP (mmHg)	0.20 (ns)	0.08 (ns)	0.01 (ns)	0.14 (ns)	**0.41 (0.002)**
DBP (mmHg)	0.13 (ns)	0.04 (ns)	0.03 (ns)	0.10 (ns)	0.24 (ns)
AC (mg/m^2^)	0.03 (ns)	0.01 (ns)	0.10 (ns)	0.13 (ns)	−0.11 (ns)
Cranial RT (y/n)	**0.44 (<0.001)**	0.26 (ns)	0.22 (ns)	**0.36 (0.007)**	**0.34 (0.011)**
Mediastinal RT (y/n)	0.02 (ns)	0.09 (ns)	0.07 (ns)	0.04 (ns)	−0.11 (ns)
GH (NPX units)	**−0.38 (0.004)**	−0.27 (ns)	**−0.29 (0.036)**	**−0.36 (0.007)**	**−0.31 (0.025)**
CRP over detection limit	0.23 (ns)	0.21 (ns)	**0.36 (0.008)**	**0.40 (0.003)**	0.18 (ns)
NT-pro-BNP over cut-off	0.01 (ns)	−0.11 (ns)	−0.20 (ns)	−0.03 (ns)	0.15 (ns)
ccaDI (%/10 mmHg)	**−0.38 (0.007)**	−0.20 (ns)	0.13 (ns)	0.07 (ns)	**−0.40 (0.002)**
ccaSI	0.11 (ns)	0.10 (ns)	−0.18 (ns)	0.04 (ns)	**0.27 (0.044)**
CIMT (mm)	−0.16 (ns)	−0.11 (ns)	−0.06 (ns)	−0.01 (ns)	−0.16 (ns)
LVPWd/BSA (mm/m^2^)	−0.10 (ns)	**−0.37 (0.007)**	−0.16 (ns)	**−0.28 (0.038)**	−0.04 (ns)
LVEF (%)	−0.09 (ns)	−0.08 (ns)	−0.11 (ns)	−0.14 (ns)	0.04 (ns)
TDI é (cm/s)	−0.09 (ns)	−0.09 (ns)	−0.14 (ns)	−0.13 (ns)	0.10 (ns)
TAPSE (mm)	−0.15 (ns)	−0.15 (ns)	−0.21 (ns)	**−0.27 (0.046)**	−0.17 (ns)
GLS (%)	0.06 (ns)	0.11 (ns)	0.18 (ns)	0.26 (ns)	0.11 (ns)

*r* denotes correlation coefficient; *P*-values are shown in parentheses.

BMI, body mass index; HR, heart rate; SBP, systolic blood pressure; DBP, diastolic blood pressure; AC, cumulative anthracycline dose; RT, radiotherapy; GH, growth hormone; CRP, C-reactive protein; NT-pro-BNP, N-terminal-pro-brain natriuretic peptide; DI, distensibility index; SI, stiffness index; CCA, common carotid artery; CIMT, carotid intima-media thickness; LVPWd/BSA, left ventricular posterior wall thickness in diastole indexed to body surface area; LVEF, left ventricular ejection fraction; TDI *é*, tissue Doppler-derived *é*-wave velocity; TAPSE, tricuspid annular plane systolic excursion; GLS, global longitudinal strain; NPX, normalized protein eXpression. *P*-values <0.05 were considered signifcant and are written in bold text.

C18:0 was correlated with BMI (*r* = 0.37, *P* = 0.008). Heart rate was correlated with C16:0, C18:0, C24:1, and CERT2 score (*r* = 0.26–0.36, *P* ≤ 0.038). Systolic blood pressure was correlated with CERT2 score (*r* = 0.41, *P* = 0.002).

There were no significant correlations of ceramides or CERT2 score with cumulative AC dose or mediastinal RT. Cranial RT was correlated with C16:0 (*r* = 0.44, *P* < 0.001) and with C24:1 and CERT2 score (*r* = 0.34–0.36, *P* ≤ 0.011). Growth hormone was correlated with C16:0 (*r* = −0.37, *P* = 0.007), C24:1(*r* = −0.34, *P* = 0.012), and CERT2 score (*r* = −0.31, *P* = 0.025). C-reactive protein was correlated with C24:0 and C24:1 (*r* = 0.36 and *r* = 0.40, respectively, *P* ≤ 0.008).

Further, there was a correlation of ccaDI with C16:0 and with CERT2 score (*r* = 0.29 and *r* = 0.40, respectively, *P* ≤ 0.029). Left ventricular posterior wall thickness in diastole was correlated with C18:0 and C24:1 (*r* = −0.37 and *r* = −0.28, respectively, *P* ≤ 0.038). Tricuspid annular plane systolic excursion was weakly correlated with C24:1 (*r* = −0.27, *P* ≤ 0.048). Global longitudinal early diastolic strain rate was weakly correlated with C24:1 (*r* = −0.27, *P* = 0.043).

**Figure 3:**
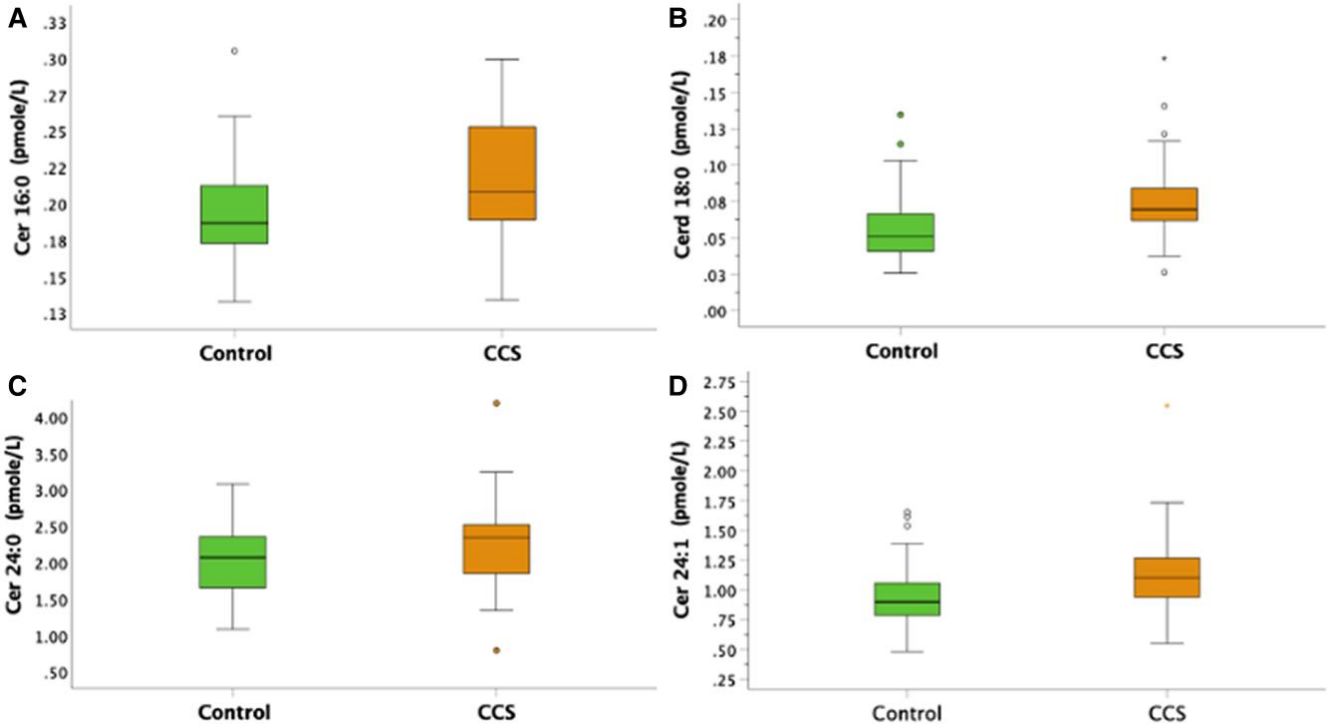
Sub-analysis of different cardiotoxic ceramides between childhood cancer survivors not exposed to cranial radiotherapy, *n* = 47, and controls, adjusting for sex, age, and body mass index. Differences in cardiotoxic ceramides between childhood cancer survivors and controls after removing childhood cancer survivors treated with cranial radiotherapy adjusted for sex, age, and body mass index (ANCOVA). All ceramides were significantly higher in childhood cancer survivors. (*A*) *P* = 0.002, (*B*) *P* < 0.001, (*C*) *P* = 0.032, and (*D*) *P* < 0.001.

Because cranial RT and GH were significantly correlated with ceramides and CERT2 score, we performed a sub-analysis of ceramides, removing CCS exposed to cranial RT from the comparison to controls, adjusting for sex, age, and BMI (*Figure 3A–D*). All ceramides were still significantly higher in CCS compared with controls in this sub-analysis. Further, correlations of GH to ceramides were still significant for some ceramides after removing CCS exposed to cranial RT (C16:0, *r* = −0.40, *P* = 0.006, C18:0, *r* = −0.24, *P* = 0.11 (ns), C24:0, *r* = −0.28, *P* = 0.07 (ns), C24:1, *r* = −0.33, *P* = 0.03).

## Discussion

To our knowledge, this is the first study of ceramides associated with CVD in CCS. We show, in the current study, that ceramides associated with CVD are markedly increased in this population compared with controls. Since all four ceramide subtypes studied herein (C16:0, C18:0, C24:0, and C24:1) have previously been shown to be strong predictors of MACE including death in the general population and in different CVD cohorts, we suggest that ceramides could be a potential circulating cardiovascular biomarker for CVD screening and primary prevention in CCS.^9,11,22,23^

The causal link between ceramides and CVDs such as atherosclerosis and heart failure has been suggested in studies in humans and animal models.^4,6,24^ Ceramides are important for cancer treatment efficacy because of their cell-growth-inhibiting and pro-apoptotic functions. Cancer treatment could possibly increase ceramide production.^25–27^ Circulating ceramides accumulate in cardiovascular tissues and cause dysfunction by increasing lipid uptake, inhibiting glucose uptake, and further by inducing inflammation, oxidative stress, and mitochondrial dysfunction leading to cellular senescence and apoptosis, processes intricately linked to the progression of CVDs.^28^

Ceramides have also been implicated in insulin resistance, a precursor to conditions such as diabetes, further amplifying cardiovascular risk.^29^ Through their influence on lipid metabolism and cellular signalling cascades, ceramides contribute to a milieu conducive to atherosclerosis and plaque formation.^3,30^ Lipid accumulation in blood vessels leads to local ceramide synthesis in endothelial cells. These locally derived ceramides cause increased levels of reactive oxygen species and activation of NADPH-oxidase, which decreases nitric oxide availability resulting in impairment of vasodilation and endothelial dysfunction.^31^

Increased levels of circulating ceramides are mainly caused by *de novo* synthesis due to lipid excess. Despite the very low concentrations compared to lipoproteins and apolipoproteins, ceramides exhibit properties that have heavy implications in CVD.^3,28^ Childhood cancer survivors are more prone to dyslipidaemia, and we believe this is the main reason for the observed high levels of ceramides in the studied CCS cohort.^32^ The reason for dyslipidaemia in CCS has been suggested to be lower physical fitness because of cardiotoxic treatments and disabilities, lower physical activity, and unhealthy diet.^33^

Combined with the damage to the cardiovascular system from chemo- and radiotherapy, accumulation of cardiotoxic ceramides in CCS might be important for the increase in risk for CVD in CCS. The studied CCS cohort was young (20–30 years) and ceramides might be an important early cardiovascular biomarker in this population for prevention strategies. Circulating ceramide levels can be lowered by up to 30% with lipid-lowering medications such as statins and PCSK9 inhibitors that also are effective in lowering LDL cholesterol.^7,34^ These treatments, including the effect of diet and exercise, should be studied regarding ceramides and the possibility to decrease the high cardiovascular morbidity and risk in CCS.^35^

In the current study, C18:0 was 33% higher in the CCS cohort compared with controls. Higher levels of circulating C18:0 have been shown to be associated with MACE in patients without known CVD independently of other cardiovascular risk factors and with an increased risk for death upon admission for heart failure.^11,36^ C18:0 has also been suggested to be a mediator for incident Type-2 diabetes, which is an important risk factor for CVD.^37^ The correlation of C18:0 to BMI in our study could suggest adiposity in CCS as a possible cause of this difference. C18:0 in skeletal muscle has been shown to cause insulin resistance in obese mice, and deficiency of the enzyme (ceramide-synthase-1—CerS1) producing C18:0 in skeletal muscle has been shown to be protective of insulin resistance in different studies.^38,39^ Whether BMI-lowering lifestyle modifications and novel enzyme inhibitors for ceramide synthases might contribute to normalization of ceramide levels in CCS are important questions that need to be addressed in future studies.^40,41^

The correlation of C18:0 and C24:1 with a lower left ventricular posterior wall thickness (i.e. thinner heart wall) is somewhat intriguing since cardiac accumulation of ceramides has earlier been reported to be associated with ventricular hypertrophy (in rodent models).^29^ Previous treatment with cardiotoxic anthracyclines causing reduced heart mass and thinner walls due to apoptosis with subsequent progression to a restrictive or dilated cardiomyopathy in some patients (the Grinch Syndrome) is likely the explanation of this apparent contradiction.^42^

In a previous study by Nwabuo *et al.*^43^ including 2700 individuals with a mean age of 66 years from the Framingham Offspring Study, the ratio of C16:0 to C24:0 was low to moderately correlated (0.3 < *r* < 0.4) with different measures of both systolic and diastolic left ventricular function (LVEF, global circumferential strain, and left atrial end-systolic volume). They concluded that this finding could explain one pathophysiological mechanism of the link between ceramides and risk for heart failure. In the current study, we found a weak correlation between C24:1 and a lower TAPSE, supporting a possible detrimental effect of ceramides on the myocardial systolic function.^43^ It is possible that the young age of the studied CCS cohort in our study precludes more significant correlations with impairments of cardiac measures since accumulation of cardiotoxic ceramides is likely a continuous phenomenon.

Ceramide accumulation has been implicated in the development of acute AC cardiomyopathy due to apoptosis signalling.^25^ A recent study in rats showed that anthracyclines increase ceramides in serum and blocking ceramide production reduces AC cardiotoxicity.^26^ Also, in another recent study using human fibroblast cell lines, anthracyclines caused the accumulation of C16:0–C24:1 resulting in inflammation with secondary apoptosis and fibrosis.^27^ However, in the current study, we found no correlations of the cumulative dose of AC treatment with any ceramides suggesting that either the effect is not dose-dependent or it is not applicable in humans.

Cranial RT is known to cause cardiometabolic complications in CCS, mainly through GH deficiency.^44^ In the current study, cranial RT was correlated with higher levels of C16:0 and C24:1, whereas higher GH was correlated with lower levels of ceramides and lower CERT2 score in CCS. Growth hormone deficiency is not exclusive to CCS that underwent cranial RT, and it has been described after chemotherapy treatment.^45^ In our sub-analysis, we showed that ceramides were increased in CCS compared with controls independently of cranial RT. This suggests that the herein observed increased levels of circulating ceramides could in part be driven by lower levels of GH not caused by cranial RT. Growth hormone deficiency can be targeted by replacement therapy, which is used only in selected cases in CCS because of risk for secondary malignancies.^44^

Ceramides are elevated in obese adults and have been shown to mediate oxidative stress and inflammation.^46^ In the current study, we found correlations of ceramides C24:0 and C24:1 with CRP, supporting a link between an inflammatory state and ceramides in CCS.

Broad ceramide panels are still difficult to use in clinical practice. Therefore, ceramide risk tests have been developed. The CERT2 is based on quartiles of four ratios of circulating C16:0, C18:0, C24:0, and C24:1 and two phospholipid species and yields a score between 0 (best) and 12 (worst).^10^ Coronary event risk test 2 has been validated for adults aged above 40 years and independently predicts 10-year risk for fatal and non-fatal cardiovascular events and is currently in use in clinical practice.^10^ Coronary event risk test 2 score can be used for CVD risk estimation in patients with and without known coronary disease.^11,22,23^

It is therefore of interest that, in the current study, the CCS cohort showed 43% higher CERT2 scores, with 35% of CCS having a CERT2 score > 7 (defined as high between 7 and 8 and very high between 9 and 12 points) compared with 9.4% of controls. Using CERT2 score charts developed by Hilvo *et al.*,^21^ the CVD risk among adults with a score of >7 points is significantly increased, with relative risks ranging between 3 and 48 compared to persons with a low (0–3 points) CERT2 score. In the current study, the investigated CCS cohort included young adults aged 20–30 years, with no burden of overt CVD. Although the CERT2 score is not adapted for this age group, the CERT2 data raise an important question whether increased ceramide levels could contribute to the elevated CVD risk in CCS later in their life.

The Childhood Cancer Survivorship Study’s CCS-specific CVD-risk calculator, developed by Chow *et al.*,^47^ accounts for exposure to cardiotoxic cancer treatments as well as diabetes and dyslipidaemia. Using this calculator, the CCS in the current study exhibits relative risks for heart failure and myocardial infarction multifold to that of siblings. It might be reasonable to suggest that ceramides and the CERT2 score might be a complement to the current risk assessment in young adult CCS because CVD occurs earlier in CCS and modifiable risk factors are even more important in CCS than in the general population.^16,48^

Our data show that the CERT2 score was correlated with a higher SBP. The CERT2 score and C16:0 were correlated with a higher HR and an increase in arterial stiffness. C16:0 has been implicated as one of the key drivers of atherosclerosis by its uptake into the endothelial cell mitochondria membranes where they form channels and cause cytochrome-c release and induction of apoptosis.^49^ Further, ceramides cause endothelial dysfunction by decreasing nitric oxide (NO) production and by increasing NO breakdown by the formation of reactive oxygen species.^31^ These data suggest that a combination of elevated circulating levels of these ceramides, perhaps driven by C16:0, might cause an increase in vascular tone coincident with increased arterial stiffness.

## Limitations

The cross-sectional design precludes conclusions regarding the predictive role of the studied ceramides and elevated CERT2 scores in clinical CVD. Other measures of the metabolic syndrome like measures of insulin resistance and liver function would have given a better insight into the pathophysiology of cardiotoxic ceramides in CCS. The large number of performed statistical tests increases the risk for Type I errors in shown correlations.

## Conclusion

In this first study in young adult CCS of ceramides associated with CVD risk and pathophysiology, we show markedly elevated serum concentrations of cardiotoxic ceramides (C16:0, C18:0, C24:0, and C24:1) in CCS compared with controls. It is reasonable to suggest that these ceramides might be important biomarkers in understanding the pathophysiology of CVD in CCS. Ceramides and the CERT2 score could possibly be important for CVD risk assessment in CCS beyond the exposure to cardiotoxic treatments.

## Supplementary Material

oeae026_Supplementary_Data

## Data Availability

The data analysed in this study are available upon request from the corresponding author (O.B., olof.broberg@med.lu.se).

## Lead author biography

Dr Olof Broberg is a paediatric cardiologist working with interventional paediatric cardiology at the Pediatric Heart Center, Skåne University Hospital, Lund, Sweden. He studied medicine at Copenhagen and Lund Universities and completed his paediatric residency at Skåne University Hospital. He has done a fellowship in paediatric interventional cardiology at Schneider Children´s Medical Center, Petah Tikva, Israel. Under the mentorship of P.L., he is doing research in cardiovascular disease after treatment for childhood cancer.
