# Supplementary material for: Ceramides: a potential cardiovascular biomarker in young adult childhood cancer survivors?
Source: Eur Heart J Open. 2024 Apr 4;4(2):oeae026. doi: 10.1093/ehjopen/oeae026 (PMC11042783; doi:10.1093/ehjopen/oeae026)
Supplement: oeae026_Supplementary_Data [file oeae026_supplementary_data.pdf]

Supplement 1. Baseline Characteristics and Oncologic Treatments in CCS of different Childhood Cancer Diagnoses

The median follow-up time of the whole cohort was 16.41 years after ending treatment. The most common diagnosis in the cohort was leukemia (n=23) followed by lymphoma (n=19).

Leukemia patients consisted of three survivors of acute myeloblastic leukemia (AML) and 20 survivors of acute lymphoblastic leukemia ALL). Lymphoma survivors consisted of 11 survivors of Hodgkin's disease (HD) and 8 survivors of non-Hodgkin's lymphoma (NHL, T- and B-cell). In the sarcoma group there were four rhabdomyosarcomas, one osteosarcoma and one Ewing's sarcoma survivors.

The highest cumulative doses of AC were in the sarcoma group (median dose 293 mg/m^2, range 197 – 302). The highest dose of total AC was given in the lymphoma group (471 mg/m^2).

In total 23 patients had received RT. The most common type was mediastinal RT followed by cranial RT. Cranial RT was only performed in leukemia patients. Mediastinal RT was given to lymphoma patients, and in one Wilms patient. The other types of RT were local RT for sarcoma treatment and abdominal RT for Wilms disease patients.

Steroid treatments consisted of either dexamethasone or prednisone and was most common in leukemia and lymphoma patients. Asparaginase was most common in leukemia patients.

S1. Summary of the Different Childhood Cancer Diagnoses and Treatments in the CCS cohort.

Diagnosis	Total n=58	Leukemia, n=23	Lymphoma, n=19	Wilms, n=10	Sarcoma, n=6
Age (years)	25.0 (22.0 –30.0)	26.0 (22.0 – 30.0)	24.1 (22.1 – 29.6)	25.1 (22.0 –29.0)	23.5 (22.1 – 28.0)
FUT (years)	16.0 (6.1 – 26.9)	19.4 (7.5 – 26.9)	10.4 (6.1 – 22.8)	21.1 (16.4 –26.5)	15.9 (12.7 – 23.5)
Sex (females, %)	23 (40.0 %)	8 (34.8 %)	7 (36.8%)	5 (50.0%)	3 (50.0%)
Anthracycline (n, %)	53 (91.%)	23 (100.0%)	19 (100.0%)	5 (50.0%)	6 (100.0%)
Cum Anthracycline dose (mg/m2)	186.5 (0 – 471.3)	219.6 (120 – 446)	160 (97 – 471.3)	25 (0 – 200)	293.4 (197 – 302)
Cyclophosphamide (n, %)	30 (52.6%)	11 (47.8%)	16 (84.2%)	2 (20.0%)	1 (16.7%)
Steroid (n, %)	36 (63.2%)	20 (87.0%)	15 (79.0%)	0 (0.0%)	1 (16.7%)
Asparaginase (n, %)	17 (29%)	15 (65.2%)	2 (10.5%)	0 (0.0%)	0 (0.0%)
Methotrexate (n, %)	26 (45.6%)	21 (91.3%)	5 (26.3%)	0 (0.0%)	0 (0.0%)
Dactinomycin (n, %)	14 (24.7%)	0 (0.0%)	0 (0.0	9 (90.0%)	6 (100.0%)
Cytarabine (n, %)	25 (44.0%)	20 (87.0%)	5 (26.3%)	0 (0.0%)	0 (0.0%)
Ifosfamid (n, %)	22 (38.6%)	10 (17.5%)	8 (42.1%)	0 (0.0%)	5 (83.3%)
Etoposide (n, %)	17 (29.8%)	10 (17.5%)	13 (68.4%)	0 (0.0%)	5 (83.3%)
Bleomycin (n, %)	1 (1.8%)	0 (0.0%)	1 (5.3%)	0 (0.0%)	0 (0.0%)
Dacarbazine (n, %)	6 (10.5%)	0 (0.0%)	6 (31.6%)	0 (0.0%)	0 (0.0%)
Procarbazine (n, %)	5 (8.8%)	0 (0.0%)	5 (26.3 %)	0 (0.0%)	0 (0.0%)
Platinum Agents (n, %)	3 (3.5%)	0 (0.0%)	0 (0.0%)	2 (20.0%)	1 (0.0%)
Vinca Alkaloids (n, %)	51 (89.5 %)	23 (91.3%)	16 (79.0%)	10(100.0%)	6 (100.0%)
RT (y/n)	24 (42.1%)	8 (31.6%)	9 (47.4%)	4 (40.0%)	3 (50.0%)
Mediastinal RT (n, %)	10 (17.5%)	0 (0.0%)	9 (47.4%)	1 (10.0%)	0 (0.0%)
Cranial RT (n, %)	8 (14.0%)	8 (14.0%)	0 (0.0%)	0 (0.0%)	0 (0.0%)

Treatments according cancer diagnosis. Number and (%) are shown. Age, follow-up time after treatment, and the cumulative anthracycline dose is shown as the median and range. **Abbreviations:** AC – anthracyclines, Cum AC – cumulative anthracycline dose, CPh – cyclophosphamide, RT – radiotherapy, FUT – follow-up time.

S2 – Ceramides, Phospholipids, Ratios and the CERT1 and CERT2 scores in CCS and Controls.

	CCS (n=57)	Controls (n=53)	P-value
C16:0 (pmol/L)	0.23 (0.05)	0.19 (0.04)	**<0.001**
C18:0 (pmol/L)	0.08 (0.03)	0.06 (0.02)	**<0.001**
C24:0 (pmol/L)	2.29 (0.58)	2.02 (0.48)	**0.012**
C24:1 (pmol/L)	1.20 (0.34)	0.95 (0.27)	**<0.001**
PC22:6 (pmol/L)	0.59 (0.36)	0.52 (0.24)	0.25
PC16:0 (pmol/L)	12.27 (2.53)	11.35 (2.04)	0.052
PC22:5 (pmol/L)	36.69 (7.79)	34.58 (7.13)	0.092
C16:0/C24:0	1.02 (0.24)	0.10 (0.02)	0.37
C18:0/C24:0	0.035 (0.01)	0.028 (0.01)	**0.006**
C24:1/C24:0	0.53 (0.11)	0.48 (0.09)	**0.007**
C16:0/PC22:5	0.006 (0.002)	0.006 (0.001)	0.058
C18:0/PC24:6	0.17 (0.10)	0.12 (0.06)	**0.006**
CERT1-score	4.42 (2.64)	3.06 (2.03)	**0.004**
CERT2-score	5.70 (2.15)	3.98 (2.01)	**<0.001**

Differences between childhood cancer survivors and controls analyzed with analysis of co-variance (ANCOVA) adjusted for age, sex and body mass index. **Abbreviations:** CCS – childhood cancer survivors, C16:0 – Cer(18:1/16:0), C18:0 – Cer(18:1/18:0), C24:0 – Cer(18:1/24:0), C24:1 – Cer(18:1/24:1), PC22:6 – PC(14:0/22:6), PC16:0 – PC(16:0/16:0), PC22:5 – PC(16:0/22:5), CERT1 – coronary event risk test 1, CERT2 – coronary event risk test 2.
